# The modern histopathologist: in the changing face of time

**DOI:** 10.1186/1746-1596-3-25

**Published:** 2008-06-06

**Authors:** Biman Saikia, Kirti Gupta, Uma N Saikia

**Affiliations:** 1 Department of Immunopathology Postgraduate Institute of Medical Education and Research, Chandigarh, India; 2 Department of Histopathology, Postgraduate Institute of Medical Education and Research, Chandigarh, India

## Abstract

The molecular age histopathologist of today is practicing pathology in a totally different scenario than the preceding generations did. Histopathologists stand, as of now, on the cross roads of a traditional 'visible' morphological science and an 'invisible' molecular science. As molecular diagnosis finds more and more applicability in histopathological diagnosis, it is time for the policy makers to reframe the process of accreditation and re-accreditation of the modern histopathologist in context to the rapid changes taking place in this science. Incorporation of such 'molecular' training viv-a-vis information communication technology skills viz. telemedicine and telepathology, digital imaging techniques and photography and a sound knowledge of the economy that the fresh entrant would ultimately become a part of would go a long way to produce the Modern Histopathologist. This review attempts to look at some of these aspects of this rapidly advancing 'art of science.'

## Introduction

In an era when we are talking of molecular classification of traditional histology and immunotherapy for cancers, the role of the histo-morphologist seems to be exploring areas which one could never have imagined a few decades ago. With the rapidly advancing field of biotechnology and molecular biology, a modern histopathologist is expected to be well versed not only in the traditional histopathological techniques but also keep pace with the ever expanding frontiers of science and technology. With molecular diagnosis threatening to overrule the histopathological diagnosis with every new discovery, it is time for the histopathologist to embrace and incorporate the recent advancements of practicing pathology in its present modern context.

With the fast changing scenario, it is but obvious that the accreditation of the histopathologist needs to undergo a drastic change, and re-accreditation of those already in the field a necessity. The current accreditation process prevalent across most of the countries is to train a student with varying degrees of vigorousity and at the end of the training period, assess the candidate through an examination which gives the license to practice pathology throughout life. In this scenario, it is totally up to the pathologist's own efforts and interest to keep up all the enthusiasm of keeping abreast with the newer technologies and new diagnostic trends. Whereas primary accreditation looks at what the new practitioner can do, re-accreditation looks at what established practitioners actually do. The accreditation and re-accreditation procedure hence needs to incorporate not only the traditional histopathological techniques but also the fields of biotechnology, telecommunication, information communication technology, professional photography and a bit economics. To this list could be added a number of other entities such as socio-psychological factors, hospital management, quality control and host of others which is beyond the scope of this discussion. The subject of accreditation and re-accreditation however has been a topic of constant reviews where authors have tried to devise list of areas where a histopathologist should be able to demonstrate continuing fitness to practice, and activities like recording educational activity, testing a pathologist's knowledge and interpretative skills, testing his diligence and peer review/appraisal has been proposed as various ways and tools to assess a practicing pathologist [1].

## Discussion

There was a time when Pathology was considered to be the mother of medical science. Histopathology, over the years has witnessed its evolution from a mainly autopsy based pathology to the current molecular histopathology. Recent advances in various fields of science and technology and the incorporation of these to histopathological practice and acceptance of some philosophical concepts, particularly the functional correlation of morphological studies, has changed the outlook of both histopathology and the histopathologist. An elaborate review on the topic at the turn of the century by Mapstone and Quirke [2] on the pathologist in context to the 21^st ^century reveals that the ideas and concepts that were envisioned then, has to a large extent, already found widespread acceptance in the pathology community, if not moved further into it. This is particularly true for the application of information technology, and to a lesser extent to molecular biology advances.

It's been sometime now since the human genome has been sequenced. The initial enthusiasm of reading the blueprint of human biology however seems nowhere in sight and it is likely to be a number of decades before many of the expected benefits of the genomics revolution are actually realized to the extent initially expected. The human genome project seems to have opened the Pandora's Genomic Box resulting in more questions than answers.

The first and most widespread application of molecular techniques in histopathology perhaps came in the form of immunohistochemistry (IHC), though IHC perhaps cannot be called a molecular technique in its true sense. Albert H. Coons and his colleagues were the first to use fluorescent dye labeled antibodies to identify antigens in tissue sections. Subsequently, enzyme labels such as peroxidase (Nakane and Pierce 1966), alkaline phosphatase (Mason and Sammons 1978) and Colloidal gold (Faulk and Taylor 1971) were introduced. Within a short time, histopathology literature was flooded with immunohistochemistry-based studies, expanding both the list of markers as well as the list of confusion. A pubmed search as on 31^st ^March 2008 for "immunohistochemistry" yielded a total of 415067 papers with 12,426 reviews reflecting the impact immunohistochemistry have had on the scientific community. The impact of the scientific breakthroughs has however started becoming more apparent gradually. This can be said more confidently when we talk of the contribution of the gene array technology, which allows gene expression measurements of thousands of genes in parallel, providing a powerful tool for pathologists seeking new markers for diagnosis. With such kind of data accumulating rapidly, it won't be long when molecular signatures would be assigned to each and every pathological lesion and a molecular diagnosis would come even before the paraffin sections are ready, and the histopathologist would already know what to look for in the sections.

Various studies have demonstrated that micro-array based gene expression profiling enables accurate tumor classification and can be very helpful diagnostic tool for cancers with unknown primaries and histologically undifferentiated tumors [3,4]. Molecular profiling has also given way to the histo-clinical classifications [5-7] since such a classification would be expected to corroborate more accurately with the functional behavior of a tumor. Molecular classification systems have been attempted extensively in organ systems like the breast and the kidney and to a lesser extent in lungs, thyroid, endometrium, ovarian, testicular, and sarcomas. Molecular classification systems in breast carcinoma and renal cell carcinoma have been shown to be of relevance in not only classification and diagnosis but also in assessing response to therapy, and as a predictor of survival [8,9]. Whereas genomic studies are establishing new molecular classifications, genetic alterations are also being identified and characterized, generating new targets for therapy and new tools to predict disease recurrence and response to therapy. This combined molecular approach is expected to have an impact on individual 'tailored' therapy for cancer patients.

However, with the concept of "tumor heterogeneity" being increasingly recognized, it has become imperative to define and analyze pure population of cells separately within the neoplasm before assigning a molecular signature to a particular neoplasm. The advent of Laser capture microdissection (LCM) technique has brought about this reliable procurement of pure population of cells from tissue sections under direct microscopic visualization and bridged a very significant technical gap between the histopathologist's microscope and the molecular biologist's work bench. This has now opened the doors to enhancing our understanding of molecular mechanisms regulating cellular developments and its functioning both in normal and diseased states.

Whereas techniques like immunohistochemistry are relatively simple and can be performed by the technical staff, more sophisticated techniques involving handling of cell culture for LCM and other molecular methods require a more intricate knowledge on the part of the pathologist for meaningful interpretation. It is thus important that a student is given hands-on experience on performing these techniques, designing of such experiments and more importantly interpreting the data, during the initial training.

The initial excitement surrounding the development of DNA micro array analysis and proteomics has also raised questions about the role of these techniques in actual clinical practice and patient management [10]. Though it is theoretically possible to build a comprehensive gene expression database for each of the organ systems/tumor type and use it as a clinical diagnostic and prognostic tool, microarray technology remains complex and time consuming and hence till date remains largely a research tool. It is prudent thus that the histopathologist takes this molecular approach with all his background knowledge of morphology rather than leave tumor diagnosis entirely in the hands of the molecular biologist to have a better and long lasting impact.

Nature however has never been too kind to the scientist. The story does not end with mere expression or non-expression of a gene and production of the corresponding mRNA. What matters at the end is the functionality of the end product which is the 'functional' protein. The story only begins with the production of the mRNA and the probability of the mRNA ultimately landing up in a functional protein would depend on the smooth co-ordination of an even more complicated array of biological functions like splicing, post-translational modifications, DNA methylation, acetylation and a host of other epigenetic factors. What needs to be assayed is therefore the functional aspect of the gene and the focus hence shifts from genomics to proteomics and more recently to glycomics. The latter, scientists believe, will have an equally dramatic effect as genomics have had. Given that a single protein can come with 10 or more different forms of sugars attached to it, giving the protein subtle differences in function, which cannot be detected by the current techniques in proteomics, leaves us with the possibility of modulating the function of the protein by modulating the activity of the enzymes that make the sugars on the protein. With the advent of the "glyco-chip" it is now possible that histopathologists too will be hunting for the sugars on these proteins and possibly do "Immuno-glyco-histochemistry" to look at the functional aspect of the protein they currently detect with immunohistochemistry. The scope is ever expanding and unlimited.

### Information Communication Technology (ICT) and the histopathologist

Working in isolated environments where access to peers, education and information is limited, is one of the highest risk factors for physicians' loss of medical competence [11]. This can't be less true for the histopathologist as well. So it's evident that for keeping pace with all the advances taking place at an outstanding pace, dissemination of information becomes a major issue. It is not surprising therefore that medical science has found its place in the electronic media or *vice versa*. It is also not surprising that most if not all prominent journals in life sciences are available on-line, though with a price tag and majority of them has options for online-submission and peer review. Information Communication Technology has also paved way for telemedicine and a science which, a few years back was a mere concept is rapidly gaining recognition as a well defined, accepted and effective application and is already showing its impact in clinical practice and modern healthcare. While telemedicine is a wider concept, Telepathology is the sub-discipline of telemedicine that deals with the capture, transmission, and viewing of pathological and histological images via telecommunication channels such as the internet, dedicated satellite or telephone, as opposed to the conventional methods of microscopy. Offering a host of innovative benefits and applications, modern telepathology systems now help to deliver more accurate remote diagnoses and are tending to replace traditional consultancy methods using light microscopy.

Telepathology over the years has undergone drastic evolution from static telepathology, which is a store-and-forward concept to dynamic, real time telepathology, using fully motorized robotic systems. A concordance rate of as high as 99–100% has been reported between telepathology and light microscopic diagnosis using such technologies [12,13]. What has however revolutionized telepathology applications and has to a great extent removed the most fundamental drawback of telepathology, that is the limitation of the available image for diagnosis, is the concept of the "virtual slide."

Virtual slides are digitized images where the entire slide is scanned at a very high resolution, acquiring the entire image of the histopathology section at all magnifications available on the microscope. Software driven motorized stage helps acquire all the fields of view and digitally stitch all the images into one single image which can be viewed by multiple pathologists.

The novel array microscope for the first ultra-rapid virtual slide processor (D Metrix DX-40) digital slide scanner is an example [14]. The optics consists of a stack of three 80-element 10 × 8-lenset array. Uniquely shaped lenses in each of the lenslet arrays constitute a single "miniaturized microscope" constituting a set of 80 microscopes. Scanning a glass slide with the array microscope produces seamless two-dimensional image data of the entire slide i.e. a virtual slide. Image acquisition can be as rapid as 58 secs for a 2.25 cm^2 ^tissue section and 40 slides can be scanned per hour. The only limitation would be the extremely large file size of the images generated, sometimes exceeding 1.5 Gb and hence the limitation with the bandwidth available for transmitting these images. It is however not unachievable considering the fact that investing in such a facility with a high bandwidth capacity is a one time effort, the fruit of which can be reaped for a long time. So, with the advent of virtual microscopy, it may be possible that an expert referral pathologist in the near future will be interpreting virtual images on LCD screens rather than glass slides. What needs to be emphasized is incorporation of the concept right at the inception of the pathology training with hands-on experience on these concepts rather than giving a theoretical knowledge for the simple reason that the factor which has till now hampered the growth and wide-spread application of telepathology is the mindset of the traditional pathologist, despite scientific evidence of efficacy of the technique. A technology can improve only when it finds its place in routine practice, and it is only through trial and error that a desired perfection is achieved. The situation however is not that grim, and excellent examples of telepathology applications have been set even in a country like India which is fast shedding its third world image. With the opening of the School of Telemedicine at the Sanjay Gandhi Post Graduate Institute of Medical Sciences at Lucknow, the first of its kind in the country, telepathology promises a bright future in the times to come.

### Histopathologist as a professional photographer

A morphologist's job is to play with images, be it live or captured. Photography hence has been an integral part of pathology practice since its inception. But chemical photography now has largely been replaced by digital photography for obvious reasons. Digital cameras utilize charge-coupled devices (CCD) or Complementary Metal Oxide Semiconductor (CMOS) image sensors to measure light energy and their circuitry to convert the measured information into a digital signal. Digital photography has the advantage of lower running cost, instant availability without any processing, easy archiving and transmission through the electronic media. It allows for adjusting, enhancing, and annotating images. Moreover it allows for post acquisition image optimization and modification to suit one's satisfaction of image quality using softwares like Adobe Photoshop. This however also carries the disadvantage and the fear of falsification of images. But as is true for every technological advance distraught with some side-effects, the advantages of digital photography in histopathology practice far outweighs its disadvantages. Patient care is enhanced by the transmission of digital images to other individuals for consultation and education, and by the inclusion of these images in patient care documents. In research laboratories, digital cameras are widely used to document experimental results and to obtain experimental data.

Since pathology is a visual science, the inclusion of quality digital images into lectures, teaching handouts and electronic documents is as essential as taking lectures. A few institutions have gone beyond the basic application of digital images to developing large electronic atlases, animated, audio-enhanced learning experiences, multidisciplinary internet conferences, and other innovative applications. So isn't it wise for a postgraduate student to handle a short course on photography at the beginning of the training as a pathologist?

### Histopathologist and the Economy

The Indian Healthcare sector, estimated to be US$ 30 billion has been growing at a frenetic pace in the past few years. Revenues from the healthcare sector account for 5.2 per cent of the GDP and currently it employs over 4 million people. Private spending accounts for almost 80 per cent of total healthcare expenditure. The burgeoning healthcare market and recent government initiatives [15] accompanied by the socio-economic changes in the population has made the Indian healthcare industry an attractive investment proposition.

The private sector already accounts for about 70% of India's health care services market and this private healthcare will continue to be the largest component in 2012 and is likely to double to US$ 35.7 billion [16]. The chances thus of a newly qualified pathologist of landing up in the private sector and contributing to its growth is perhaps as high as, if not more, than the chances of being in the public sector.

#### The Histopathology Market

The series of technologies developed over the years to address the challenges of molecular diagnostics include eight major areas: amplification technologies (gene and signal), micro array technologies, blotting technologies, electrophoretic technologies, probe technologies, restriction fragment-length polymorphism (RFLP) analysis, RNA inhibition analysis, and single nucleotide polymorphism (SNP) analysis with significant overlap among them to support one another. These developments have set the stage for excellent growth potential in the marketplace. Histopathology, an approximately 1 billion market worldwide is growing at 10% per annum [17] despite the fact that molecular diagnostic's contribution to such statistics is only a minuscule. Further down the century, histopathology, molecular biology and IT sector combined will make the modern Histopathologist's market enjoy a good share in the healthcare sector. Is the fresh entrant into pathology training aware of this growing aspect of healthcare?

## Conclusion

So, are we looking at a histopathologist, who is not only a good traditional morphologist, but also an IT savvy scientist, with drops of Leonardo Da Vinci and Picasso in blood, who is ready to take technology head-on, without the inhibitions the current generation of pathologists face and who would look at the histopathology market with a much wider perspective of the current economy? The making of this Modern Histopathologist would require a drastic change in the accreditation procedure and a major initiative on the part of the policy makers and the teachers of Pathology. The transition from a field of visual interpretations to a largely invisible molecular science will take some time to firm foot but will eventually be there.

## Competing interests

The authors declare that they have no competing interests.

## Authors' contributions

BS and KG conceptualized, designed and drafted the manuscript. UNS did the critical revision for important intellectual content. All authors read and approved the final manuscript.
